# Sanders’ New Books

**Published:** 1906-09

**Authors:** 


### SANDERS' NEW BOOKS.

Messrs. W. B. Sanders Company announce for publication in the early fall the following excellent and practical works:

Keen's Surgery—Its Principles and Practice (Volume I.).
Sobotta and McMurrich's Human Anatomy (Volume III.).
Webster's Text-Book of Gynecology.
Hill's Histology and Organography.
McConnell's Pathology.
Morrow's Immediate Care of the Injured.

Stevenson's Photoscopy (Retinoscopy and Skiascopy).

Preiswerk and Warren's Atlas of Dentistry.

Goepp's State Board Questions and Answers.

Lusk's Elements of Nutrition.

The most notable announcement is the new work on Surgery, edited by Dr. W. W. Keen, complete in five octavo volumes, and containing over 1,500 original illustrations. The entire work is written by the leaders of modern surgery—men whose names are inseparably associated with the subjects upon which they have written. Without question, Keen's Surgery will represent the best surgical practice of to-day.

---

### X-Ray Diagnosis of Kidney Stone.

Holland sums up his paper as follows: When a stone or stones are present in such size as to produce symptoms suggesting the desirability of operation, if a careful and thorough examination is made such stone or stones can nearly always be shown by X-rays. And this would also apply to the presence of stone even if the symptoms alone were not sufficient to demand operation. In most cases when shadows are shown the size, shape and position can be relied on in diagnosing them as from kidney or ureteral stones. In other cases when doubt may arise as to the cause of the shadows the experience of the examiner will often settle the matter, and in some cases stereoscopic radiography, the use of ureteral bougies, etc., can be used as a help. The negative diagnosis can be relied on only when the whole region on both sides is carefully examined and when the plates are taken under the essential condition for successful examination and when they are of the necessary quality in showing sufficient differentiation of the soft structures in the kidney and ureteral regions. There can be no justification for operation or prolonged medical treatment without an efficient X-ray examination being made.—*Lancet, June 2, 1906.*

---

In all cases of torticollis, examine for caries of the spine.—*American Journal of Surgery.*

